# Is there hidden genetic variability in the species of *Steindachneridion* Garavello, 2005 (Siluriformes: Pimelodidae)?

**DOI:** 10.7717/peerj.18193

**Published:** 2025-03-28

**Authors:** Daniele Aparecida Matoso, Hallana Cristina Menezes da Silva, Augusto Luiz Ferreira Júnior, Fábio Porto-Foresti, Ricardo Utsunomia, Fernanda Dotti do Prado, Roberto Ferreira Artoni

**Affiliations:** 1 Departamento de Genética, Universidade Federal do Amazonas, Manaus, Amazonas, Brazil; 2 Programa de Pós-Graduação em Genética, Conservação e Biologia Evolutiva, Instituto Nacional de Pesquisas da Amazônia, Manaus, Amazonas, Brazil; 3 Programa de Pós-Graduação em Genética Evolutiva e Biologia Molecular, Universidade Federal de São Carlos, São Carlos, São Paulo, Brazil; 4 Departamento de Ciências Biológicas, Universidade Estadual Paulista Júlio de Mesquita Filho, Bauru, São Paulo, Brazil; 5 Centro de Ciências Biológicas, Universidade Estadual do Norte do Paraná, Bandeirantes, Paraná, Brazil; 6 Departamento de Biologia Estrutural, Molecular e Genética, Universidade Estadual de Ponta Grossa, Paraná, Brazil

**Keywords:** Genetics Variability, *Steindachneridion*, Brazilian basins, Haplotype network, *Steindachneridion scriptum*, *Steindachneridion melanodermatum*, *Steindachneridion parahybae*, *Steindachneridion doceanum*, D-loop region, Phylogentic variation

## Abstract

**Background:**

The genus *Steindachneridion*, which includes large-sized freshwater pimelodid species, is endemic to the southeastern coastal drainages of South America, specifically the Paraná River and Uruguay River basins.

**Methods:**

In this study, genetic analyses of mitochondrial DNA (mtDNA) D-loop were conducted on four species within this genus across their respective distributions: *Steindachneridion scriptum* (from the Tibagi and Uruguay rivers), *S. melanodermatum* (from the Iguaçu River), *S. doceanum* (from the Doce River), and *S. parahybae* (from the Paraíba do Sul River). *Zungaro zungaro* and *Brachyplatystoma rousseauxii* were employed as outgroups, and the topology was inferred using Bayesian Inference (BI) and maximum likelihood (ML) phylogenetic reconstruction techniques. Additionally, the sequences were analyzed to assess genetic diversity levels.

**Results:**

In contrast to the remaining species, which exhibited distinct species-specific clades, our data suggests that *S. scriptum* formed two sister clades, potentially representing distinct operational taxonomic units. Novel haplotypes were identified for each of the four species, further supporting the conclusions derived from the phylogenetic analysis. Overall, *Steindachneridion* species displayed high haplotype diversity paired with low nucleotide diversity, indicating a demographic expansion event after reduced effective population size. Nevertheless, genetic structure indexes were notably high. These findings suggest that the genetic diversity within these species may be underestimated, which has implications for both taxonomic classification and biological conservation strategies.

**Conclusion:**

In conclusion, the study of genetic diversity in four *Steindachneridion* species has revealed distinct molecular operational taxonomic units (MOTUs), which highlights the necessity for conservation efforts. The detection of new haplotypes and intraspecific variability emphasizes the urgency of implementing systematic conservation measures in the face of looming extinction threats.

## Introduction

The large-sized pimelodid fish genus *Steindachneridion* is a fish genus endemic to the eastern Brazilian watersheds and can also be found in the rivers of the Paraná and Uruguay basins (Garavello, 2005). Although its classification within the Pimelodidae is well accepted, its evolutionary history and ancestry remain largely unknown (Albert & Reis, 2011). Despite the lack of resolution regarding its phylogenetic relationships, it is regarded as a basal group for the superfamily Pimelodoidea (Sullivan, Muriel-Cunha & Lundberg, 2013).

In terms of taxonomy, six species are recognized under the genus *Steindachneridion* (Garavello, 2005). The fossils of the *S. iheringi* species were first reported by Woodward (1898) and dated to the Oligocene (Lima, Salard-Cheboldaeff & Suguio, 1985). This indicates that the species first emerged around 13.5 million years ago, which is compatible with the diversification of pimelodid fish.

Some studies employing molecular markers have questioned the existence of a hidden diversity within this genus. However, morphological taxonomy recognizes some interspecific diversity in *Steindachneridion* (Matoso, 2009; Matoso et al., 2011; Paixão, Ribolli & Zaniboni-Filho, 2018). For *S. parahybae*, a species on the verge of extinction (Honji et al., 2017), the use of microsatellite markers and gene sequences from the mitochondrial DNA controlling region indicated population structure due to fragmentation and anthropogenic impacts. However, an unrooted haplotype network analysis employing D-loop sequences separately grouped the species *S. scriptum*, *S. parahybae*, and *S. melanodermatum*, which exhibited no signs of population organization (Souza-Shibatta et al., 2021).

Indeed, access to mitochondrial DNA has proven to be a crucial tool for evolutionary studies and the conservation of fish species. Analysis of mitochondrial DNA provides valuable insights into the evolutionary history, genetic diversity, and population structure of aquatic organisms, helping to identify speciation patterns, migration routes, and environmental adaptations. The use of mitochondrial DNA is particularly important for the assessment and monitoring of endangered fish populations, facilitating the implementation of effective conservation strategies and sustainable management practices (Antoniou & Magoulas, 2014).

As indicated by ICMBio/MMA, *Steindachneridion* species have frequently appeared at the top of Red Book lists of endangered species since 2013 (ICMBio, 2018). This is due to a number of urgent issues, including overfishing, habitat degradation and fragmentation, which are considered to be the primary causes of the decline and extinction of freshwater fish species (Paixão, Ribolli & Zaniboni-Filho, 2018). In light of the aforementioned circumstances, it is of paramount importance to define significant taxonomic units for conservation purposes and to map population structure and genetic variability. These sources of information serve as a basis for the formulation of effective management strategies with regard to the restructuring and/or maintenance of populations and species of *Steindachneridion*. To better understand the phylogenetic and demographic relationships of this important group of Neotropical fish, this article aims to present new information concerning molecular genetic variability.

## Materials & Methods

### Sampling and D-loop PCR

**Figure 1:**
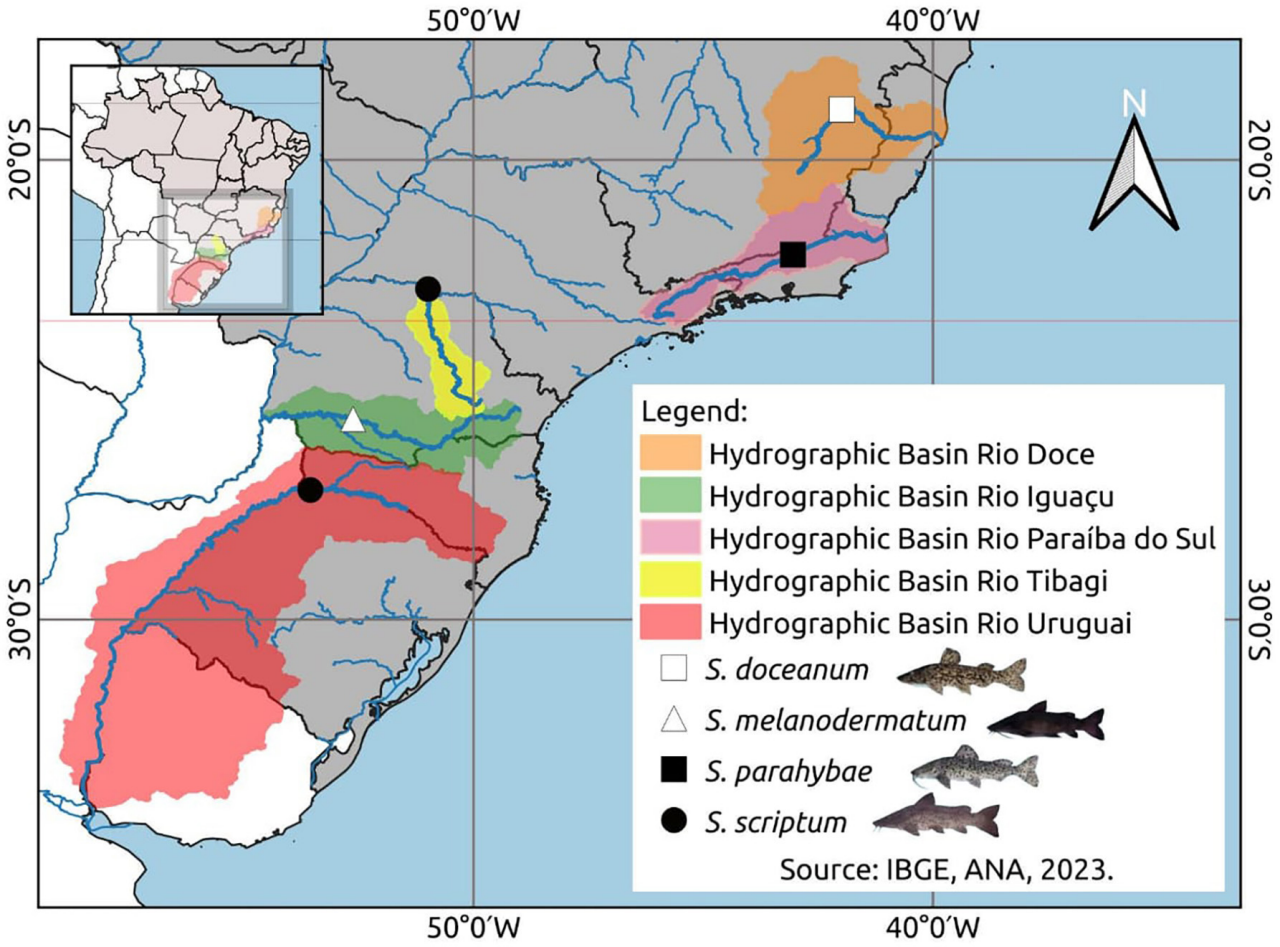
Geospatial analysis of *Steindachneridion* species distribution across South American river basins. Data on the distribution of animals in the basons was mapped using IBGE, ANA, 2023. Map and fish images sources: the authors.

Muscle samples were collected for the DNA assessment: ten *Steindachneridion scriptum* specimens from Rio Uruguay, six from Rio Tibagi, seventeen *S. melanodermatum* specimens from Rio Iguaçu, three *S. parahybae* specimens from Rio Paraíba do Sul, and two *S. doceanum* specimens from Rio Doce (Fig. 1), following the procedures outlined by Sambrook, Fritsch & Maniatis (1989).

The amplification of the D-loop region was conducted using five ng of DNA, 1.5 mM MgCl2, 1x buffer, 200 mM of each dNTP, 0.5 U of Taq DNA polymerase, and 0.5 mM of each primer (FTTF: 5′CAA AGC GCC GGT CTT 3′ and F12R 5′GTC AGG ACC ATG CCT TTG3′) (Sivasundar, Bermingham & Ortí, 2001). PCR was initiated with five cycles, comprising denaturation at 94 °C for one minute, hybridization at 53 °C for one minute, and elongation at 72 °C for one minute and 30 s, followed by 25 cycles at 50 °C for the hybridization step. PCR reactions were conducted using a PTC-100 thermocycler from MJ Research (Waltham, MA, USA). PCR products were subjected to electrophoresis in a 0.8% agarose gel utilizing phage l as the molecular mass marker. Samples were purified using the GFX PCR DNA and Gel Band Purification Kit (GE HealthCare, Chicago, IL, USA), followed by sequencing using the DYEnamic ET Terminator Cycle Sequencing kit (GE HealthCare) as per the manufacturer’s instructions. Bidirectional sequencing of all templates was carried out on a Sanger ABI 3130 sequencer (Applied Biosystems, Waltham, MA, USA), with images documented by the Kodak Electrophoresis Analysis and Documentation System (EDAS) 290. Sequences of *Steindachneridion scriptum* from Rio Uruguay and Rio Tibagi (accession numbers EU930030–EU930044), *S. melanodermatum* from Rio Iguaçu (accession numbers PQ871622–PQ871638), *S. parahybae* from Rio Paraíba do Sul (accession numbers PQ871639–PQ871641), and *S. doceanum from* Rio Doce (accession numbers PQ871620–PQ871621) were deposited in GenBank.

This research followed the international standards of animal experimentation, approved by the Ethics Committee for Animal Use of the Universidade Estadual de Ponta Grossa (CEUA process number 0769342/2021). The collection was authorized by the Ministério do Meio Ambiente (MMA/ICMbio number 15115-1).

### Genetic diversity analysis

Genetic distances within and between groups were estimated based on the Kimura 2-parameter evolution model (K2P) (Kimura, 1980). A neighbor-joining (NJ) tree (Saitou & Nei, 1987) was constructed from this model to represent divergences between species to depict species divergences, graphically, and clade support was assessed by 1,000 bootstrap pseudo-replicates according to (Felsenstein, 1985), using MEGA 10.2.2 (Kumar et al., 2018). Genetic diversity parameters (*S*—polymorphic sites; *Nh*—number of haplotypes; *h*—haplotypic diversity; *π*—nucleotide diversity) were evaluated utilizing DnaSP 6.12.03 (Rozas et al., 2017) and employed neutrality tests including Tajima’s D (Tajima, 1989), Fu’s Fs (Fu, 1997) and *R*_2_ (Ramos-Onsins & Rozas, 2002).

Statistical significance was determined *via* 10,000 permutations under the coalescing process implemented in DnaSP 6.12.03 (Rozas et al., 2017). To elucidate relationships between species of the genus *Steindachneridion* and the population of *Steindachneridion melanodermatum* studied, haplotype networks were reconstructed using PopART 1.7 (Leigh & Bryant, 2015) through the TSC method (Clement, Posada & Crandall, 2000).

### Phylogenetic analysis of mtDNA D-loop sequences

For the phylogenetic analyses, we retrieved a D-loop sequence from *Brachyplatystoma rousseauxii* with accession number DQ779046 (Batista & Alves-Gomes, 2006) and another sequence from *Zungaro zungaro* (accession number EU930046.1), as an outgroup from GenBank. These *D-loop* sequences underwent alignment and editing using the Clustal W program (Thompson, Higgins & Gibson, 1994). Subsequently, a Bayesian Inference (BI) analysis was conducted employing MrBayes v. 3.2.7 (Ronquist & Huelsenbeck, 2003) through Markov chain Monte Carlo (MCMC) searches on two simultaneous runs of four chains for 10^6^ generations, sampling trees every 10^3^ generations. A burn-in of 25% of the sampled trees was implemented, and consensus topology and nodal support were computed from the remaining trees, estimated as posterior probability values (Huelsenbeck & Ronquist, 2001). Visualization and finalization of phylogenetic trees were executed using FigTree v. 1.4.4 (http://tree.bio.ed.ac.uk/software/figtree/). Additionally, the PAUP* program (Swofford, 2002) was utilized to determine the best evolutionary model *via* Modeltest 3.7 (Posada & Crandall, 1998) under the hierarchical likelihod ratio tests (hLRTs). The maximum likelihood (ML) analysis was performed using MEGA 11.0.13 (Tamura et al., 2007), employing the HKY model with a gamma distribution, and bootstrap resampling with 501 replications to assess node support.

## Results

### Genetic diversity of *Steindachneridion*

Nucleotide sequencing retrieved a total of 38 partial *D-loop* sequences of *Steindachneridion scriptum* samples collected from the Rio Uruguay and Rio Tibagi. The calculated average nucleotide diversity across these sequences was 0.065, and *S. scriptum* specifically exhibited a nucleotide diversity of 0.017. Comparing of the nucleotide diversity and haplotype count within *S. scriptum* populations revealed that the Uruguay River harbored a greater number of haplotypes (*N* = 10). In contrast, the Tibagi River displayed higher nucleotide diversity (*N* = 6).

For *S. doceanum*, the determined nucleotide diversity was 0.030, while *S. melanodermatun* exhibited a nucleotide diversity of 0.011. Notably, a nucleotide diversity of 0.130 was observed in *S. parahybae*. The calculated *R*_2_ values were substantially higher and statistically significant, indicating evidence of a departure from neutrality. However, in contrast, the Fu’s (*Fs*) test metric showed relatively high values, which, despite their magnitude, did not achieve statistically significance.

It is important to note that the *R*_2_ test has been acknowledged as more effective in detecting demographic events in smaller samples sizes (Ramos-Onsins & Rozas, 2002), as observed in the present study. Conversely, Fu’s (*Fs*) test tends to be more suitable for samples with more of individuals, as emphasized in the comparison presented in Table 1.

**Table 1:** Genetic diversity characterization for *Steindachneridion* fishes (Pimelodidae) in South America.

**Sampling**	***N***	***n***	***S***		***Nh***	***h ± SD***	***π ± SD***		***D***	***Fs***	***R***_2_
*Steindachneridion (Total-mean)*	38	861	237		36	0.997 ± 0.007	0.07254 ± 0.01656		−1.69962^ns^	0.444^ns^	0.162^**^
*S. scriptum*	16	849	58		15	0.992 ± 0.025	0.01668 ± 0.00217		−1.07389^ns^	0.450^ns^	0.162^**^
Tibagi River	6	843	25		6	1.000 ± 0.096	0.01205 ± 0.00220		−0.54595^ns^	0.451^ns^	0.162^**^
Uruguai River	10	849	29		9	0.978 ± 0.054	0.00777 ± 0.00158		−1.90383^*^	0.439^ns^	0.162^**^
*S. melanodermatum*					
Iguaçu River	17	846	43		16	0.993 ± 0.023	0.01093 ± 0.00156		−1.33687^ns^	0.437^ns^	0.162^**^
*S. doceanum*					
Doce River	2	814	24		2	1.000 ± 0.500	0.03011 ± 0.01506		n/c	0.363^ns^	0.161^**^
*S. parahybae*					
Paraíba do Sul River	3	658	117		3	1.000 ± 0.272	0.13060 ± 0.04295		n/c	0.348^ns^	0.161^**^

**Notes.**

*N* number of individuals examined *n* total number of sites *S* number of segregating sites *Nh* number of haplotypes *h* haplotype diversity *π* nucleotide diversity *D* Tajima’s neutrality test *Fs* Fu’s neutrality test *R* neutrality test [21] *SD* Standard Deviation n/c not calculated

ns not significant (*p* > 0.05).

* Significant (*p* < 0.05).

** Highly significant (*p* < 0.01).

**Figure 2:**
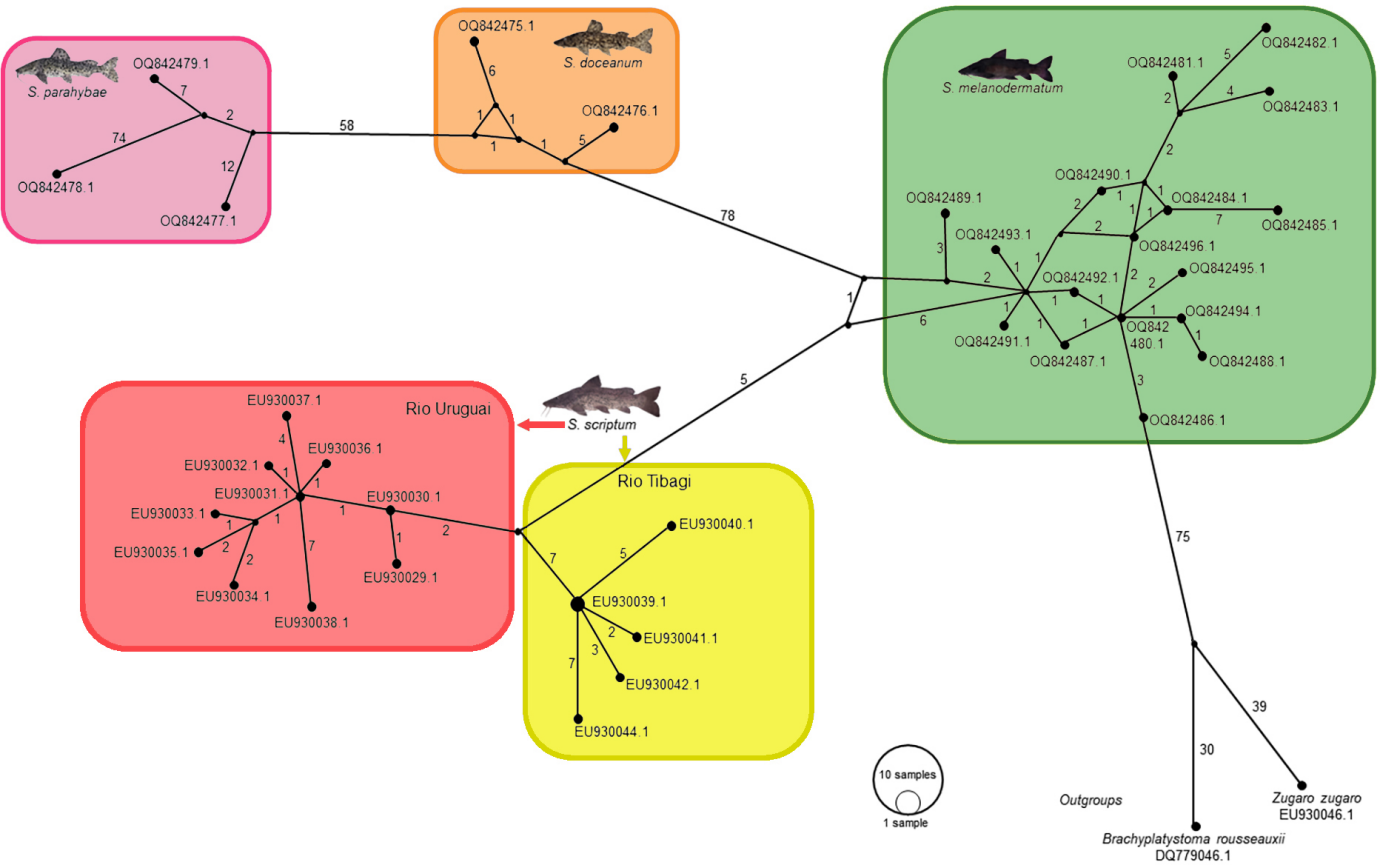
Haplotype network depicting mitochondrial D-loop gene variation among *Steindachneridion* species (Pimelididae) from South America. Haplotypes are represented for each species and their respective river basins: *S. doceanum* from the Rio Doce basin (orange); *S. parahybae* from the Rio Paraiba do Sul basin (pink); *S. scriptum* from the Rio Tibagi and Rio Uruguay basins (yellow and red, respectively); *S. melanodermatum* from the Rio Iguaçu basin (green). The outgroup species are depicted in black: *Zungaro zungaro*; *Brachyplatystoma rousseauxii* (haplotype D31). Fish images source: the authors.

**Figure 3:**
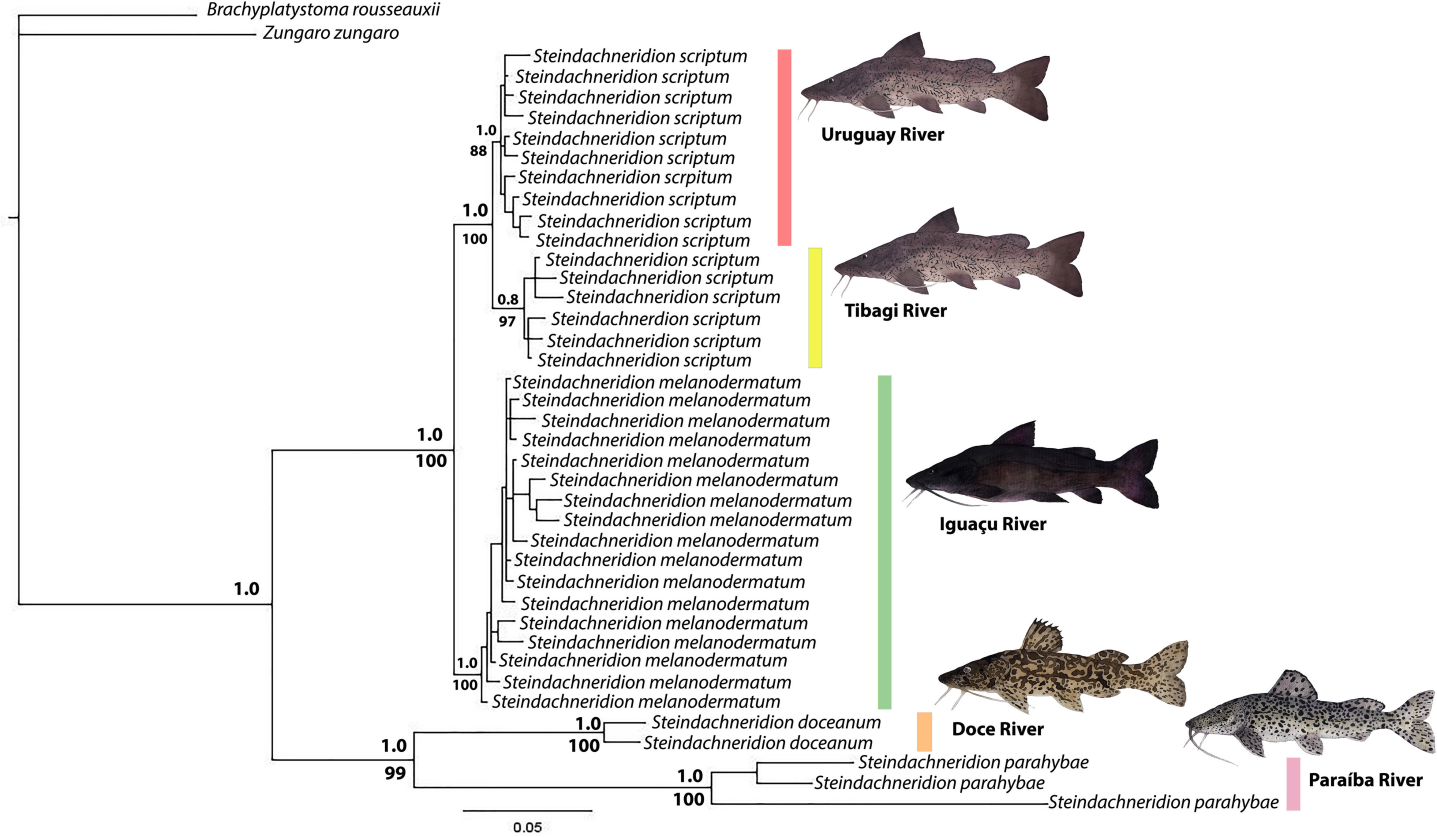
Phylogenetic topology and genetic distances (HKY model) among *Steindachneridion* species sourced from South American rivers utilizing mitochondrial D-loop gene data. The outgroup species are represented in black: *Zungaro zungaro*; *Brachyplatystoma rousseauxii* (haplotype D31). Fish images source: the authors.

In the context of haplotype composition, our analysis revealed the identification of novel haplotypes within different species. Specifically, two new haplotypes (Haplo 1 and 2) were identified in *S. doceanum*, while three distinct haplotypes (Haplo37, Haplo38, and Haplo39) were found in *S. parahybae*. Moreover, a total of fifteen haplotypes were detected in *S. scriptum*, and sixteen haplotypes were observed in *S. melanodermatum*, labeled as Haplo28 to Haplo44 (as depicted in Figs. 2–3).

In the haplotype network analysis, a closer relationship was observed between *S. scriptum* populations from Rio Tibagi and Rio Uruguay, with only nine mutational steps separating these two populations. Comparatively, when contrasting *S. melanodermatum* with *S. scriptum* populations from the Uruguay River and Tibagi River, 13 and 18 mutational steps were identified, respectively. In addition, a considerable distance was found between *S. parahybae* and the other species, with 72 mutational steps from *S. parahybae* to *S. melanodermatum* and 61 mutational steps to *S. doceanum*.

Regarding the outgroup species, the analysis revealed significant genetic distances. Specifically, between *S. melanodermatum* and the outgroup species, *Brachyplatystoma rousseauxii* and *Zungaro zungaro*, 104 and 113 mutational steps were observed, respectively. These findings provide insights into the genetic divergence and relationships among the studied species, emphasizing notable genetic distances and relationships within and between species.

### Phylogenetic analysis

**Figure 4:**
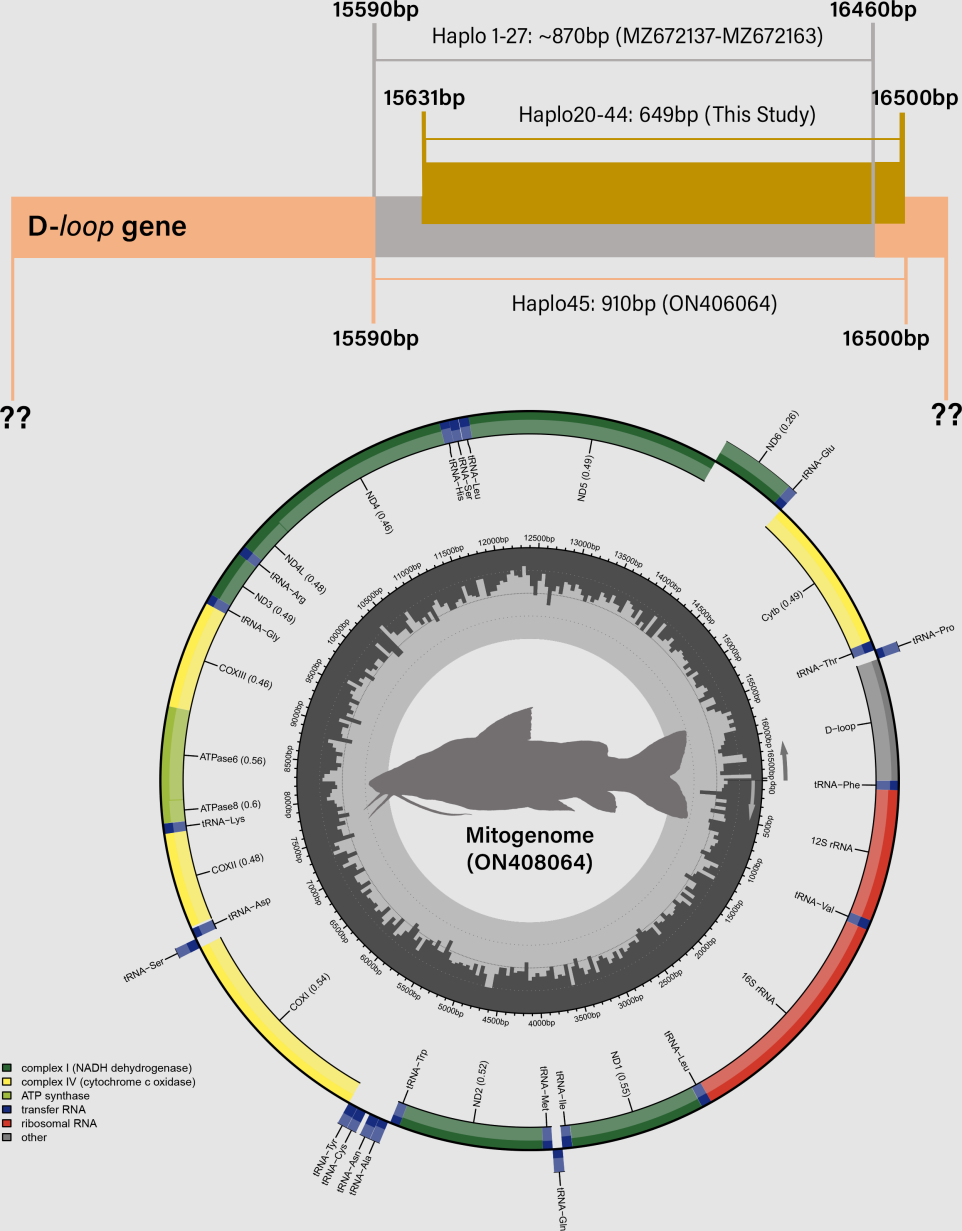
Distribution of mitochondrial D-loop gene barcodes among *Steindachneridion melanodermatum* samples sourced from South American rivers, alongside sequences available in GenBank mitogenomes (accession ON408064). Base pair (bp) positions are indicated. Haplotypes are designated as Haplo1 to 27—MZ672137 to MZ672163 (refer to Souza-Shibatta et al., 2021) and Haplo28 to 44—PQ871620–PQ871638 (from this study). GenBank accessions include Haplo1—ON408064. Fish images source: the authors.

The topology resulting from the analyses using the BI and ML methods showed significant similarities (Fig. 4). The genus *Steindachneridion* was determined to be monophyletic for the four species examined in this study, grouped in a highly robust clade supported by a high posterior probability. *Steindachneridion scriptum* from the Tibagi and Uruguay rivers exhibited consistent clustering into two distinct clades, supported by bootstrap and posterior probability values. *Steindachneridion doceanum* grouped with *S. parahybae*, while *S. melanodermatum* formed a single clade. The species *B. rousseauxii* and *Z. zungaro* clustered together, comprising the sister group to all the analyzed species.

### Annotation and characterization of haplotypes of *S. melanodermatum*

**Figure 5:**
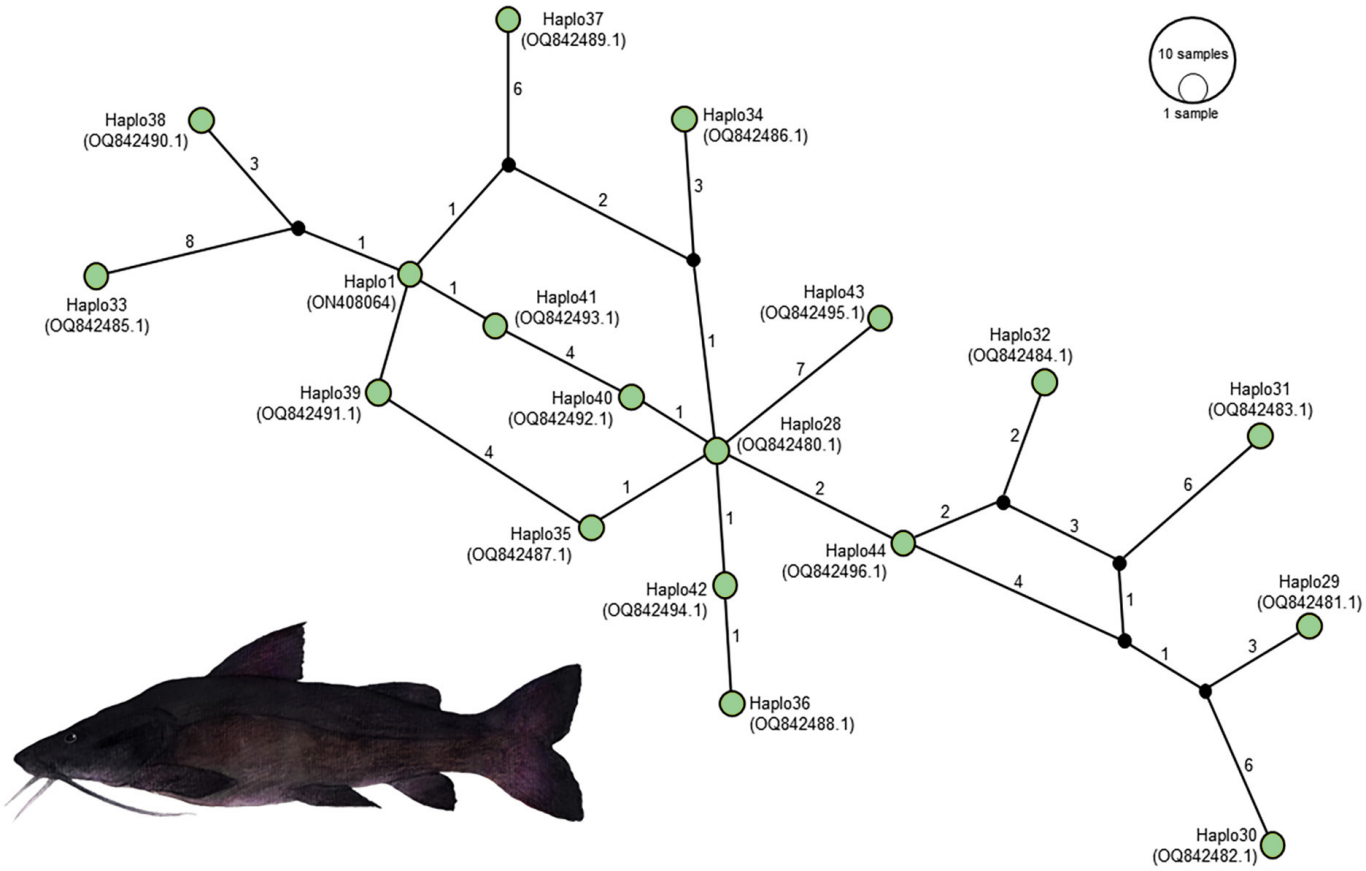
Haplotype network illustrating the mitochondrial D-loop gene variation in *Steindachneridion melanodermatum* (S.m, *n* = 18) sourced from South American regions. The identified haplotypes range from Haplo28 to Haplo44 (sequences PQ871620–PQ871638, obtained in this study), alongside GenBank accessions such as Haplo1—ON408064. Haplotypes not detected are represented by black circles. Fish images source: the authors.

Partial sequencing of the mitochondrial *D-loop* gene resulted in an array of 849 base pairs, situated between positions 15,651 and 16,500 within the mitogenome of *S. melanodermatum* (ON408064) (Fig. 5). The 27 additional *D-loop* haplotypes (Haplo1 to Haplo27) retrieved from references MZ672137–MZ672163 encompassed positions 15,590 to 16,460 within the mitogenome (Haplo1), constituting a sequence length of 870 base pairs (depicted in Fig. 3).

Alignment of these obtained sequences with those available in GenBank revealed an overlap spanning 809 base pairs (from positions 15,651 to 16,460), flanked by two non-overlapping fragments situated upstream (positions 15,590 to 15,650) and downstream (positions 16,461 to 16,500) the overlapping region (as illustrated in Fig. 4). Notably, these non-overlapping fragments represent non-conserved regions within the sequences and did not influence the identification of *S. melanodermatum* haplotypes.

This comprehensive alignment and analysis clarify the specific regions used for sequence comparisons, underscoring the conserved nature of certain segments and their role in facilitating the accurate identification of *S. melanodermatum* haplotypes despite non-overlapping fragments.

## Discussion

### Genetic diversity unveiled: a glimpse into *Steindachneridion* is enigmatic variability

The investigation of genetic diversity among the four *Steindachneridion* species has revealed a clustering mosaic of haplotypic variation, exhibiting substantial diversity levels (*h* = 0.97–1.00—Table 1). It is noteworthy that our methodologies, which are analogous to those employed in studies on *S. scriptum* from the Uruguay River (Paixão, Ribolli & Zaniboni-Filho, 2018) and *S. melanodermatum* from the Iguaçu River (Souza-Shibatta et al., 2021), are in agreement with the findings of these investigations. This revelation assumes particular intrigue given the status of these species as endemics (Garavello, 2005), which are believed to inhabit deep wells and are facing imminent extinction threats (ICMBio, 2018).

The integration of haplotype network data with information obtained from phylogenetic analysis, conducted through BI and ML, revealed the presence of two distinct evolutionary units for the species *Steindachneridion scriptum*. These molecular operational taxonomic units (MOTUs) were identified in the Tibagi and Uruguay rivers, respectively (Figs. 1 and 3). This finding is consistent with prior research conducted in the Upper Uruguay River (Paixão, Ribolli & Zaniboni-Filho, 2018). The presence of substantial genetic structure, coupled with evidence of departure from neutrality (as indicated by the R2 statistic and Fs statistic’s results, respectively), underscores the complexity within these populations.

Remarkably, discovering of numerous new haplotypes across the four *Steindachneridion* species signals an untouched reservoir of genetic variability within the genus. While previous studies, such as the one by Fonseca et al. (2017), employing microsatellite markers, revealed low heterozygosity rates in *S. parahybae*, the conundrum of how intrapopulation genetic variability is maintained in *Steindachneridion* remains unresolved.

The emerging kinship dynamics among *Steindachneridion* species paint a clearer picture. Our haplotype network asserts a robust genetic affinity between *S. melanodermatum* and *S. scriptum* from the Uruguay River. These findings echo the conclusions drawn by Souza-Shibatta et al. (2021), who, though employing different methodologies (six microsatellite markers in a larger sample size (*N* = 95)), reveled analogous connections between these species. Despite *S. melanodermatum*’s seemingly uncompromised genetic diversity, clues pointing to a recent bottleneck were evident in microsatellite data (Matoso et al., 2011). This study expands on previous knowledge, revealing 16 novel haplotypes for *S. melanodermatum*, illuminating the pressing need for deeper investigations into the species’ genetic diversity.

Our discovery of several new haplotypes within the studied *Steindachneridion* species underscores the potential undervaluation of their genetic diversity. The cohesive groupings observed in the haplotype network, coupled with BI and ML analyses, accentuate each species’ distinctiveness as a single operational taxonomic unit (OTU), warranting due consideration for conservation efforts. Additionally, our findings, aligning with those by Fonseca et al. (2017) in *S. parahybae*, highlight the conundrum posed by the combination of low nucleotide diversity and high haplotypic diversity, hinting at a demographic surge following a period of diminished effective population size.

### Unraveling evolutionary enigmas: insights into *Steindachneridion* is the genetic tale

The investigation of morphological data and the genetic model data from nuclear and mitochondrial sources has revealed a remarkable evolutionary history within the Pimelodidae family (Lundberg & Littmann, 2003). This history is evidenced by the existence of a monophyletic clade (Sullivan, Muriel-Cunha & Lundberg, 2013). Similarly this monophyly is supported for the species of *Steindachneridion* (Garavello, 2005), which places these fish at the base of the pimelodid lineage (Lundberg, Sullivan & Hardman, 2011). Such evidence calls for a deeper comparative and kinship investigation, particularly within the *Steindachneridion* genus. Leveraging BI and ML analyses on 38 mitochondrial *D-loop* gene sequences from our studied species yielded robust clades, providing high reliability indices (Fig. 3). This analytical exploration was made possible by the comprehensive mitochondrial genome of *S. melanodermatum* (Silva et al., 2024), which was pivotal for concatenation of nucleotide sequences in our phylogenetic algorithms for BI and ML (Fig. 4).

Our investigations, rooted in the topology of the Pimelodidae (Lundberg & Akama, 2005), showcased *Brachyplatystoma rousseauxii* and *Zungaro zungaro* as outgroups, delineating the phylogenetic relationships among examined *Steindachneridion* species, distinctly positioned outside these clusters. Internally, these clusters identified within the four *Steindachneridion* species under scrutiny stand as conclusive evidence, reverberating uniform biogeographic patterns.

The geological dynamics of the Guiana and Brazilian continental shields and the frainage patterns along South America’s east coast have significantly shaped the river systems (Ribeiro, 2006; Lundberg, 1998). Notably, macrodome uplift, rifts, vertical movements, and erosive actions along the east bank have been pivotal in shaping the distribution of freshwater ichthyofauna in these regions. The observed biogeographic patterns (Ribeiro, 2006) underscore taxa with endemic coastal distributions maintaining phylogenetic ties with neighboring ichthyofauna, hinting at *Steindachneridion is* integration within this context. These fish, harboring endemic distributions across coastal basins (three of their six species) while spanning the Upper Paraná basins, including the Iguaçu River and Uruguay River, exhibit kinship connections with *Zungaro*, *Sorubim*, *Pseudoplatystoma*, and the Amazon basin-dwelling *Brachyplatystoma*.

The observed clustering between *S. doceanum* + *S. parahybae* and *S. melanodermatum* + *S. scriptum* could be attributed, in part, to biogeographic features along Brazil’s east coast, perhaps associated with the proximity of these species’ river headwaters. Comparable findings in the 16S gene sequences of *Hoplias malabaricus* (Dergam et al., 2002) unveiled intertwined relationships across basins like Doce, Paraíba do Sul, and Grande, remnants of a shared past during the Plio-Pleistocene. The ichthyofauna of these basins is currently a reflection of a relict fauna that still bears similarities to one another (Torres et al., 2008; Menezes et al., 2008). Erosive retreats and headwater capture events might have sculpted the ichthyofauna relationships across these basins (Ribeiro, 2006).

*Steindachneridion* is included in the National List of Aquatic Vertebrates and Fish Threatened with Extinction since 2013 (ICMBio, 2018) highlighting its conservation urgency. Species like *S. punctatum* and *S. amblyurum*, absent in our study due to sampling difficulties, emphasize their precarious status and the challenges in preserving them.

Since the diversification of Siluriformes in the Neotropical region came before the division of South America from other continental blocks (Pinna, 1998). Thus, comparative data with taxa beyond the Neotropical region are imperative to achieve a more comprehensive phylogenetic and biogeographic scenario. Future research on Sorubiminae’s systematics promises to elucidate the phylogenetic intricacies of basal lineages within this group, potentially fortifying conclusions derived herein.

Four of the six known *Steindachneridion* species were central to our study. *S. punctatum*, unfortunately, is no longer found in the wild. Beyond contributing to the evolutionary narrative of this endangered endemic genus, our primary aim was to unveil the latent genetic variability within these species. Our crucial takeaway underscores distinct MOTUs among *Steindachneridion* species, advocating for their conservation. Remarkably, recent events might have influenced genetic differentiation, hinting at intraspecific variability suggestive of speciation processes, demanding systematic consideration, as exemplified in the case of *S. scriptum*.

## Conclusions

In conclusion, the study of genetic diversity within the *Steindachneridion* genus has revealed a wide range of variability, as evidenced by the presence of distinct MOTUs among species. This discovery of genetic intricacies provides insight into the evolutionary dynamics within this group of fish and emphasizes the urgent need for conservation efforts. As we continue to explore the enigmatic evolutionary history of *Steindachneridion*, further research is essential to fully comprehend the phylogenetic intricacies and biogeographic context of these basal lineages within the Pimelodidae family. Moreover, the identification of novel haplotypes and the detection of intraspecific variability indicate the ongoing processes of speciation. This underscores the necessity for a systematic approach and proactive conservation measures, particularly in view of the imminent extinction risks faced by these endemic species.

## Supplemental Information

10.7717/peerj.18193/supp-1 Supplemental Information 1 List of sequences deposited at GenBank

## Additional Information and Declarations

### Field Study Permissions

The following information was supplied relating to field study approvals (i.e., approving body and any reference numbers): The collection was authorized by the Ministério do Meio Ambiente (MMA/ICMbio number 15115-1).

### DNA Deposition

The following information was supplied regarding the deposition of DNA sequences: For the phylogenetic analyses, we retrieved a D-loop sequence from *Brachyplatystoma rousseauxii* (DQ779046, Batista & Alves-Gomes, 2006) and another sequence from *Zungaro zungaro* (EU930046.1), as an outgroup from GenBank.

### Data Availability

The following information was supplied regarding data availability: The sequences of *Steindachneridion scriptum* from Rio Uruguay and Rio Tibagi (EU930030–EU930044), *S. melanodermatum* from Rio Iguaçu (PQ871622–PQ871638), *S. parahybae* from Rio Paraíba do Sul (PQ871639–PQ871641), and *S. doceanum* from Rio Doce (PQ871620–PQ871621) are available at GenBank.

## References

[ref-1] Albert JS, Reis RE (2011). Introduction to neotropical freshwaters. Historical biogeography of neotropical freshwater fishes.

[ref-2] Antoniou A, Magoulas A (2014). Application of mitochondrial DNA in stock identification. Stock identification methods.

[ref-3] Batista JS, Alves-Gomes JA (2006). Phylogeography of *Brachyplatystoma Rousseauxii* (Siluriformes - Pimelodidae) in the Amazon Basin Offers Preliminary evidence for the First Case of “Homing” for an Amazonian Migratory Catfish. Genetics and Molecular Research.

[ref-4] Clement M, Posada D, Crandall KA (2000). TCS: a computer program to estimate gene genealogies. Molecular Ecology.

[ref-5] Dergam JA, Paiva SR, Schaeffer CE, Godinho AL, Vieira F (2002). Phylogeography and RAPD-PCR variation in *Hoplias Malabaricus* (Bloch, 1794) (Pisces, Teleostei) in Southeastern Brazil. Genetics and Molecular Biology.

[ref-6] Felsenstein J (1985). Confidence limits on phylogenies: an approach using the bootstrap. Evolution.

[ref-7] Fonseca FS, Domingues RR, Hallerman EM, Hilsdorf AWS (2017). Genetic diversity of an imperiled neotropical catfish and recommendations for its restoration. Frontiers in Genetics.

[ref-8] Fu Y-X (1997). Statistical tests of neutrality of mutations against population growth, hitchhiking and background selection. Genetics.

[ref-9] Garavello JC (2005). Revision of genus *Steindachneridion* (Siluriformes: Pimelodidae). Neotropical Ichthyology.

[ref-10] Honji RM, Tolussi CE, Caneppele D, Polaz CNM, Hilsdorf AWS, Moreira R.G (2017). Biodiversity and conservation of ichthyofauna threats of extinction in the River Basin Paraíba Do Sul. Revista da Biologia.

[ref-11] Huelsenbeck JP, Ronquist F (2001). MRBAYES: bayesian inference of phylogenetic trees. Bioinformatics.

[ref-12] ICMBIO (2018). Red book of Brazilian fauna threat of extinction.

[ref-13] Kimura M (1980). A simple method for estimating evolutionary rates of base substitutions through comparative studies of nucleotide sequences. Journal of Molecular Evolution.

[ref-14] Kumar S, Stecher G, Li M, Knyaz C, Tamura K (2018). MEGA X: molecular evolutionary genetics analysis across computing platforms. Molecular Biology and Evolution.

[ref-15] Leigh JW, Bryant D (2015). POPART: full-feature software for haplotype network construction. Methods in Ecology and Evolution.

[ref-16] Lima MR, Salard-Cheboldaeff M, Suguio K (1985). Palynological Study of Tremembé training; Tertyary of the Taubaté Basin (State of São Paulo; Brazil); Dontextolês Survey Samples; 42 From the Conselho Nacional de Petroleo.

[ref-17] Lundberg JG, Malabarba LR, Reis RE, Vari RP, Lucena ZMS, Lucena CAS (1998). The temporal context for the diversification of neotropical fishes. Phylogeny and classification of neotropical fishes.

[ref-18] Lundberg JG, Akama A (2005). *Brachyplatystoma capapretum*: a new species of goliath catfish from the amazon basin, with a reclassification of allied catfishes (Siluriformes: Pimelodidae). Copeia.

[ref-19] Lundberg JG, Littmann MW, Reis RE, Kullander SO, Ferraris CJ (2003). Family Pimelodidae: long-whiskered catfishes. Check list of the freshwater fishes of South and Central America.

[ref-20] Lundberg JG, Sullivan JP, Hardman M (2011). Phylogenetics of the South American Catfish Family Pimelodidae (Teleostei: Siluriformes) using nuclear and mitochondrial gene sequences. Proceedings of the Academy of Natural Sciences of Philadelphia.

[ref-21] Matoso DA (2009). Contribution to the genetic conservation of surubim Fish (Teleostei: Siluriformes). PhD Thesis.

[ref-22] Matoso DA, Da Silva M, Cortinhas MCS, Cestari MM, De Almeida MC, Vicari MR, Artoni RF (2011). Two genetic stocks of *Steindachneridion Melanodermatum* living in sympatry in nature and genetic variability of wild parents and F1 generation. Genetics and Molecular Research.

[ref-23] Menezes NA, Ribeiro AC, Weitzman S, Torres RA (2008). Biogeography of glandulocaudinae (Teleostei: Characiformes: Characidae) revisited: phylogenetic patterns, historical geology and genetic connectivity. Zootaxa.

[ref-24] Paixão RV, Ribolli J, Zaniboni-Filho E (2018). Genetic variation of the endangered neotropical Catfish *Steindachneridion scriptum* (Siluriformes: Pimelodidae). Frontiers in Genetics.

[ref-25] Pinna MCC, Malabarba LR, Reis RE, Vari RP, Lucena ZMS, Lucena CAS (1998). Phylogenetics relationships of neouropical siluriformes (Teleostei: Ostariophysi): historical overview and synthesis of hypotheses. Phylogeny and classification of neotropical fishes.

[ref-26] Posada D, Crandall KA (1998). MODELTEST: testing the model of DNA substitution. Bioinformatics.

[ref-27] Ramos-Onsins SE, Rozas J (2002). Statistical properties of new neutrality tests against population growth. Molecular Biology and Evolution.

[ref-28] Ribeiro AC (2006). Tectonic history and the biogeography of the freshwater fishes from the coastal drainages of Eastern Brazil: an example of faunal evolution associated with a divergent continental margin. Neotropical Ichthyology.

[ref-29] Ronquist F, Huelsenbeck JP (2003). MrBayes 3: bayesian phylogenetic inference under mixed models. Bioinformatics.

[ref-30] Rozas J, Ferrer-Mata A, Sánchez-DelBarrio JC, Guirao-Rico S, Librado P, Ramos-Onsins SE, Sánchez-Gracia A (2017). DnaSP 6: DNA sequence polymorphism analysis of large data sets. Molecular Biology and Evolution.

[ref-31] Saitou N, Nei M (1987). The neighbor-joining method: a new method for reconstructing phylogenetic trees. Molecular Biology and Evolution.

[ref-32] Sambrook LB, Fritsch EF, Maniatis T (1989). Molecular cloning: a laboratory manual.

[ref-33] Silva CF, Freitas GI, Calegari RM, Garcia Y, Utsunomia R, Artoni RF, Porto-Foresti F (2024). The complete mitochondrial genome of *Steindachneridion Melanodermatum* (Teleostei; Naluriformes; Pimelodidae) an endemic neotropical species. Mitochondrial DNA Part B: Resources.

[ref-34] Sivasundar A, Bermingham E, Ortí G (2001). Population structure and biogeography of migratory freshwater fishes (*Prochilodus*: Characiformes) in major South American rivers. Molecular Ecology.

[ref-35] Souza-Shibatta L, Ferreira DG, De Assumpção L, Shibatta OA, Sofia SH, Pini SFR, da Silva PS, Makrakis S, Makrakis MC (2021). Genetic diversity of the Surubim-Do-Iguaçu, a giant catfish species threatened with extinction: recommendations for species conservation. Diversity.

[ref-36] Sullivan JP, Muriel-Cunha J, Lundberg JG (2013). Phylogenetic relationships and molecular dating of the major groups of catfishes of the neotropical superfamily Pimelodoidea (Teleostei, Siluriformes). Proceedings of the Academy of Natural Sciences of Philadelphia.

[ref-37] Swofford DL (2002). https://www.scienceopen.com/document?vid=a59ba3b2-9972-437a-ae3b-3ad61c20edd0.

[ref-38] Tajima F (1989). Statistical method for testing the neutral mutation hypothesis by DNA polymorphism. Genetics.

[ref-39] Tamura K, Dudley J, Nei M, Kumar S (2007). MEGA4: molecular evolutionary genetics analysis (MEGA) software version 4.0. Molecular Biology and Evolution.

[ref-40] Thompson JD, Higgins DG, Gibson TJ (1994). CLUSTAL W: improving the sensitivity of progressive multiple sequence alignment through sequence weighting, position-specific gap penalties and weight matrix choice. Nucleic Acids Research.

[ref-41] Torres RA, Motta TS, Nardino D, Adam ML, Ribeiro J (2008). Chromosomes, RAPDs and evolutionary trends of the neotropical fish *Mimagoniates microlepis* (Teleostei: Characidae: Glandulocaudinae) from coastal and continental regions of the Atlantic forest, Southern Brazil. Acta Zoologica.

[ref-42] Woodward AS (1898). Considerations on some Tertiary fish from the Taubaté schists, State of S. Paulo, Brazil. Paulista Museum Magazine.

